# Extensive Countrywide Molecular Identification and High Genetic Diversity of *Haemonchus* spp. in Domestic Ruminants in Greece

**DOI:** 10.3390/pathogens13030238

**Published:** 2024-03-08

**Authors:** Konstantinos V. Arsenopoulos, Styliani Minoudi, Isaia Symeonidou, Alexandros Triantafyllidis, George C. Fthenakis, Elias Papadopoulos

**Affiliations:** 1 Laboratory of Parasitology and Parasitic Diseases, School of Veterinary Medicine, Faculty of Health Sciences, Aristotle University of Thessaloniki, 54124 Thessaloniki, Greece; arsenopo@vet.auth.gr (K.V.A.); isaia@vet.auth.gr (I.S.); 2 Laboratory of Genetics, Development and Molecular Biology, School of Biology, Aristotle University of Thessaloniki, 54124 Thessaloniki, Greece; sminoudi@bio.auth.gr (S.M.); atriant@bio.auth.gr (A.T.); 3 Veterinary Faculty, University of Thessaly, 43100 Karditsa, Greece

**Keywords:** *Haemonchus contortus*, genetic diversity, phylogenetic relationship, domestic ruminants, ITS2 sequence, ND4 gene

## Abstract

The gastrointestinal nematode parasite *Haemonchus* spp. is one of the most pathogenic parasites of ruminants, due to its blood-sucking activity, which causes large economic losses in the ruminant industry. The latest epizootiological data recorded an increase in the infection, not only in Greece but also in other countries, mainly attributed to climatic changes. The study of the population structure and the investigation of the phylogenetic relationships of *Haemonchus* spp. are essential for the understanding of its biology and epizootiology to implement appropriate control and prevention strategies. In addition, the molecular approach allows the determination of evolutionary relationships between different species of this parasite, the diverse hosts they infect, as well as the different geographic compartments from which they originate. Therefore, the aim of the present study was to identify the species of the sympatric populations of the genus *Haemonchus*, a nematode parasite infecting ruminants (sheep, goats, cattle, and buffaloes) from different regions of Greece (continental and insular) using molecular methods. At the same time, an attempt was made to identify the possible subpopulations of *Haemonchus* spp. in Greece, to investigate their phylogenetic relationships, as well as to determine the genetic diversity of each population. A total of 288 worms of the genus *Haemonchus* were processed using molecular methods; of these, 96 were collected from sheep, 96 from goats, 48 from cattle, and finally, 48 from buffaloes. A fragment of 321 base pairs of the second internal transcribed spacer (ITS2) sequence of nuclear DNA was amplified for species identification, and, after basic local alignment search tool (Blast) analysis, it was revealed that they belonged to *H. contortus*. A fragment of 820 base pairs of subunit 4 of the nicotinamide dehydrogenase (ND4) gene of mitochondrial DNA was amplified for genetic diversity analysis. The Greek mitochondrial ND4 sequences of *H. contortus* were classified into 140 haplotypes, and the values of the average nucleotide and haplotype diversity were lower compared to the respective values derived from Italy, Malaysia, the USA, and China. The phylogenetic analysis of the ND4 gene revealed a clear grouping of the Greek haplotypes when compared with Asian ones, and, at the same time, there was no profound grouping of the same haplotypes with regard to their different hosts and geographical origin within different regions of Greece. The aforementioned findings confirmed that *H. contortus* prevails in our country and can infect all species of ruminants, without geographical boundaries, when the right conditions (i.e., common grazing) are created.

## 1. Introduction

*Haemonchus* spp. is a blood-sucking nematode parasite, which was first described in 1803 by Karl Rudolphi [1]. It belongs to the superfamily Trichostrongylidae and along with *Teladorsagia* spp., they consist the most pathogenic nematode parasites infecting ruminants in the developing world [2]. It can infect all species of ruminants [1], expressing its pathogenicity through the blood feeding activity [3]. The genus *Haemonchus* includes over 10 species, within which *Haemonchus contortus* and *Haemonchus placei* are the most widespread helminths. According to Hoberg et al. [4], these two parasites are globally distributed not only in the abomasa of domestic but also in non-domestic ruminants. *H. contortus* infects mainly small grazing ruminants [5,6], while *H. placei* has been detected in the abomasa of large ruminants (i.e., cattle). Other parasitic species such as *H. longistipes* infect six species of ruminants, focusing on camels, while the rarer species, *H. mitcelli* and *H. vegliai*, infect mainly antelopes and deer, respectively [4].

*Haemonchus* spp. expresses its pathogenicity through anemia due to the blood-sucking properties and the occasional death of the infected animals. The main problem for commercial flocks is the reduction in the production of the infected animals including a decrease in growth and weight of young and adult ruminants, respectively, and the reduction in produced milk and meat, which result in severe financial losses for the farmers [7]. Its global importance is highlighted by the fact that the annual treatment cost for *H. contortus* alone has been estimated to be USD 26 million in Kenya, USD 46 million in South Africa, and USD 103 million in India [8].

The geographical location of Greece has established it as the crossroads between European, Asian, and African countries. Our culture meets different cultures as a result of human movements for various reasons (i.e., tourism, immigration, asylum seeking, wars, etc.), accompanied by animal movements and their parasites. It is well known that a significant number of ruminants has been imported in Greece from other European countries [9]. Furthermore, the varied terrain of the Greek territory is combined with many different climatic conditions from low temperatures in high altitude to high-temperature/moisture environmental conditions in many islands, affecting not only the survival but also the spread of many different species of parasites [10].

The aforementioned reasons make the identification of the nematode parasite of the genus *Haemonchus* in the Greek territory, at the species and subpopulation level, an urgent need. Previous studies, based on the morphological identification of third-stage larvae (L_3_) or adult parasites, concluded that *H. contortus* was the only species found in the abomasa of sheep and goats in Greece [11,12]. Furthermore, little information is available on the species of *Haemonchus,* which parasitize cattle herds in Greece, while nothing is known, on the same issue, in buffalo herds. The only source of information regarding the different *Haemonchus* spp. comes from Himonas [13], who morphologically identified *H. placei* in the abomasa of cattle in Greece.

Relatively little is known on the genetic diversity of many parasitic populations of domesticated ruminants, including *Haemonchus* spp. Worldwide, it is recognized that the host plays an important role in determining the genetic variability and gene flow between discrete parasitic populations [14]. A number of nematode species form relatively large gene pools within continental areas, and this has been attributed, at least in some cases, to the widespread transportation of the hosts and thus the mixing of the parasite gene pool. Regarding Greece, nothing is known about the genetic variation of *Haemonchus* populations except for a study conducted by Troell et al. [15]. These authors recorded high genetic diversity after the examination of a small number (n = 8) of adult *H. contortus* from eight different sheep of the region of Thessaloniki (Central Macedonia, Greece). Finally, phylogenetic studies of *Haemonchus* spp. collected from both cattle and buffaloes have not been conducted in Greece, until now.

Therefore, the objectives of the present study were to (i) identify the different species of the nematode parasite of the genus *Haemonchus*, originating from sheep, goats, cattle, and buffaloes from both continental and insular Greece, employing the ITS2 of nuclear ribosomal DNA, and to (ii) evaluate their genetic variation, through phylogenetic analysis, aiming at the ND4 of mitochondrial DNA.

## 2. Materials and Methods

### 2.1. Parasite Material

**Figure 1:**
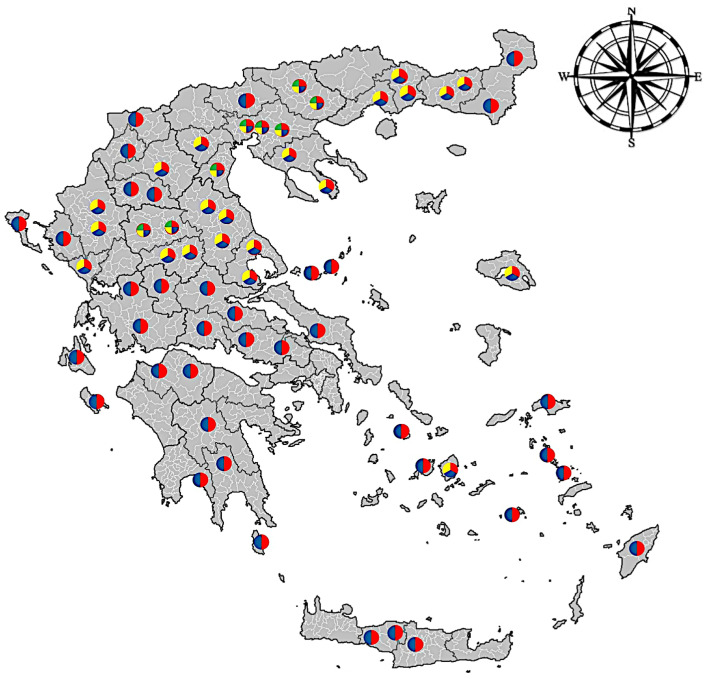
Locations of the abattoirs around Greece, which were visited for *Haemonchus* spp. sampling. The classification of the colors among four host species is as follows: red corresponds to sheep, blue to goats, yellow to cattle, and green to buffaloes.

In total, 288 individual adult female *Haemonchus* spp. were collected from the abomasa of 168 slaughtered ruminants from several local abattoirs of both continental (n = 224) and insular (n = 64) Greece (Figure 1). More precisely, 96 out of 288 parasites were collected from sheep, 96 from goats, 48 from cattle, and the remaining 48 from buffaloes. After their collection, the parasites were washed thoroughly in physiological saline and stored in containers with 70% ethanol. The samples were transported to the Laboratory of Parasitology and Parasitic Diseases of the Veterinary Medicine School of the Aristotle University of Thessaloniki (Central Macedonia, Greece).

### 2.2. Morphological Identification

After their arrival at the Laboratory, all (n = 288) *Haemonchus* spp. worms were morphologically identified according to morphological keys of spicule length and morphology and the characteristics of the pattern of the longitudinal ridges (synlophe) on the external cuticular surface of the parasite, using an optical microscope (OMAX, M82ES-SC100-LP100, Guangzhou, China) [16].

### 2.3. Isolation of Genomic DNA

After their morphological identification, all (n = 288) samples were transported to the Laboratory of Genetics, Development and Molecular Biology of the Biology School of the Aristotle University of Thessaloniki (Central Macedonia, Greece) for genomic DNA isolation. The head of each individual helminth was dissected from the rest of the body at the cervical papillae, excluding the parasite’s uterus and eggs (to avoid any genetic material shared by male parasites), and was taken for DNA extraction. Whole genomic DNA was extracted using the protocol of Hillis et al. [17], which is based on the chemical compound cetyl-trimethyl-ammonium bromide. After DNA extraction, tubes containing DNA were stored at −20 °C. The quality of the extracted DNA was evaluated using electrophoresis on 1.0% agarose gels stained with ethidium bromide and visualized using a UV transilluminator (Super-Bright UV-Pad Edge 26MX, VWR International Ltd., Lutterworth, UK).

### 2.4. PCR Amplification and Sequencing

For species identification, a fragment of 321 bp of ITS2 sequence of nuclear DNA was amplified using a pair of primers (Table 1), as described by Brasil et al. [18], produced by Eurofins Genomics GmbH (Ebersberg, Germany). For genetic diversity analysis, a fragment of 820 bp of the ND4 gene of mitochondrial DNA was amplified using a pair of primers (Table 1), as described by Troell et al. [15], produced by Eurofins Genomics GmbH (Ebersberg, Germany).

PCR amplification was performed in 30 μL reaction mixtures, containing 3 μL DNA, 15 μL KAPA Taq ReadyMix PCR kit (content: 1 unit per 50 μL reaction KAPA Taq DNA polymerase, 0.2 mM of each deoxynucleoside triphosphate [dNTP], 1.5 mM magnesium chloride and stabilizers), 0.3 μL of each primer, 1.2 μL magnesium chloride, and 10.2 μL double distilled water, in a Takara PCR thermal cycler Dice (Takara BIO Inc., Kusatsu City, Japan).

The temperature cycling protocols were the following for ITS2: 2 min initial denaturation at +94 °C; 35 cycles of strand denaturation at +94 °C for 30 s, annealing at +55 °C for 30 s, and primer extension at +72 °C for 30 s; and 10 min of final extension at +72 °C. The protocols for ND4 were as follows: 5 min initial denaturation at +95 °C; 30 cycles of strand denaturation at +95 °C for 30 s, annealing at +55 °C for 30 s, and primer extension at +72 °C for 60 s; and 5 min of final extension at +72 °C. Amplification success was assessed using electrophoresis in 1.0% (*m*/*v*) agarose gels, stained with ethidium bromide, and visualized using a UV transilluminator (Super-Bright UV-Pad Edge 26MX, VWR International Ltd., Lutterworth, UK). The PCR products were purified and sequenced using the Sanger method from Genewiz Company (Leipzig, Germany).

### 2.5. Data Analysis

The raw sequences were initially checked and analyzed using the Geneious program (version 5.1) [19] and then aligned using Clustal W [20]. The final sequences consisted of 204 bp for ITS2 and 690 bp for ND4. The number of different haplotypes for both genes and the genetic diversity (i.e., haplotype and nucleotide diversity) for the ND4 gene were determined by using the DnaSP software (version 4.50.2) [21].

For the phylogenetic analysis of the ND4 gene, two datasets were constructed. Dataset 1 included sequence data only from the present study, while Dataset 2 included Greek sequence data combined with 169 sequences from GenBank (Table 2), and upon alignment, the overlapping region was 673 bp long. A ND4 sequence of *H. placei* (accession number NC029736) was used as the outgroup.

The best fit nucleotide substitution model for these datasets was determined using jModelTest 3.7 (under the Bayesian Information Criterion, BIC) [25,26]. The Tamura–Nei model, using a discreet Gamma distribution and assuming that certain sites are evolutionary invariable (TN93 + I + G model), was used for Bayesian phylogenetic analysis, carried out with Beast 1.10.4 [27]. The Bayesian tree was constructed using a strict clock model and a “Coalescent: Constant size” tree prior. The analysis was run for 10^8^ Markov Chain Monte Carlo (MCMC) generations, sampled every 10^4^ generations. The convergence of chains was visualized using Tracer 1.7 discarding the first 20% of trees as burn-in. Effective Sample Size (ESS) values for all parameters were larger than the threshold value of 200 identified by Tracer v.1.7. The trees produced by Beast were then summarized in TreeAnnotator 1.10.4 and visualized in FigTree 1.4.3.

Additionally, one “Median-Joining” network [28] was constructed using the software Network (version 5.0.0) and the frequencies of the sequences (Fluxus Technology, Sunnyvale, CA, USA), assuming equal weights for all mutations and setting the genetic distance parameter *e* to zero to restrict the choice of feasible links in the final network. This network included the same sequences as described for Dataset 2.

### 2.6. Statistical Analysis

A chi-square test was used to check if there are any statistical differences in the distribution of the frequencies of the ITS2 genotypes among the hosts, and to indicate statistical differences of the haplotype and nucleotide diversity of ND4 gene, for Dataset 1 and 2, among the ruminant species. Data were analyzed using SPSS 23. Statistical significance was set at a = 0.05 level.

## 3. Results

### 3.1. Morphological Identification

All (n = 288) helminths morphologically evaluated during the study were fully identified as *H. contortus*.

### 3.2. Molecular Identification of Haemonchus Population, at the Species Level, Based on ITS2 Sequence

In the present study, the nuclear ITS2 sequence was successfully sequenced from 194 *Haemonchus* spp. collected from sheep (n = 74), goats (n = 63), cattle (n = 33), and buffaloes (n = 24). The identification of the nematode parasites of the genus *Haemonchus*, at the species level, was performed through molecular approaches based on the conclusions of Stevenson et al. [29] and Blast analysis. All parasites were found to belong to the species *H. contortus*. The Blast analysis of the obtained sequences showed a high nucleotide identity ranging from 99.6% to 100% with those of *H. contortus* available in GenBank.

At the same time, the possibility of the existence of heterozygous (hybrid) strains of *H. contortus* and *H. placei* was tested. This test was performed by searching for duplicate peaks in the chromatographs and specifically at positions 24, 205, and 219 of the nuclear ITS_2_ sequence. No duplicate peaks were detected at any location, which minimizes the possibility of hybridization in all samples of the present study.

The alignment and the analysis of all 194 ITS2 sequences, with the reference sequence X78803, revealed 19 genotypes (Accession numbers ON400662–ON400680) and six variable nucleotide positions (59, 63, 78, 123, 187, and 196). These substitutions represented two transversions (one G <−> T, one A <−> T) and four transitions (three C <−> T, one A <−> G). Genotypes 1 and 2 were found to be the most predominant in all four hosts. Details are presented in Table 3.

### 3.3. Estimation of Genetic Diversity of H. contortus Population Based on ND4 Gene

In the present study, the mitochondrial ND4 gene was successfully sequenced in 194 parasites collected from sheep (n = 74), goats (n = 63), cattle (n = 33), and buffaloes (n = 24), previously identified as *H. contortus*. The alignment (690 bp) of these sequences revealed 163 polymorphic sites, 118 of which were parsimony-informative. As expected for the mitochondrial gene, the base composition was rich in adenine (A) and thymine (T) at 77.4%.

The 194 sequences (Accession numbers OM401332–OM401525) were classified into 140 haplotypes, with 23 haplotypes being shared among at least two different hosts. Among the sequences from the present study (Dataset 1), the parasitic population collected from goats showed the highest haplotype diversity (0.999), while the one collected from cattle showed the lowest (0.966) value. Similarly, the highest value of nucleotide diversity is recorded in the parasitic population of sheep (0.021), while the lowest one in the respective populations of cattle and buffaloes (0.007). Moreover, haplotype and nucleotide diversity values were higher in *H. contortus* populations from small ruminants compared to total Greek samples. Finally, most (90 out of 120) haplotypes of small ruminants are unique haplotypes (host specific), while the population of *H. contortus* from goats recorded the highest percentage (66.7%) of unique haplotypes per total number of parasites, followed by the population of *H. contortus* from sheep (Table 4).

The combined analysis of the 194 sequences of *H. contortus* from Greece with the 169 sequences from GenBank (Dataset 2) revealed 306 haplotypes, of which 24 are shared between at least two different hosts. In this dataset, a higher nucleotide diversity value was recorded in the sheep and goat (0.029) parasitic populations as well as in the total number of samples (0.026). Finally, more than 80% of sheep and goat haplotypes were unique haplotypes (Table 4).

### 3.4. Phylogenetic Analysis of ND4 Gene

**Figure 2:**
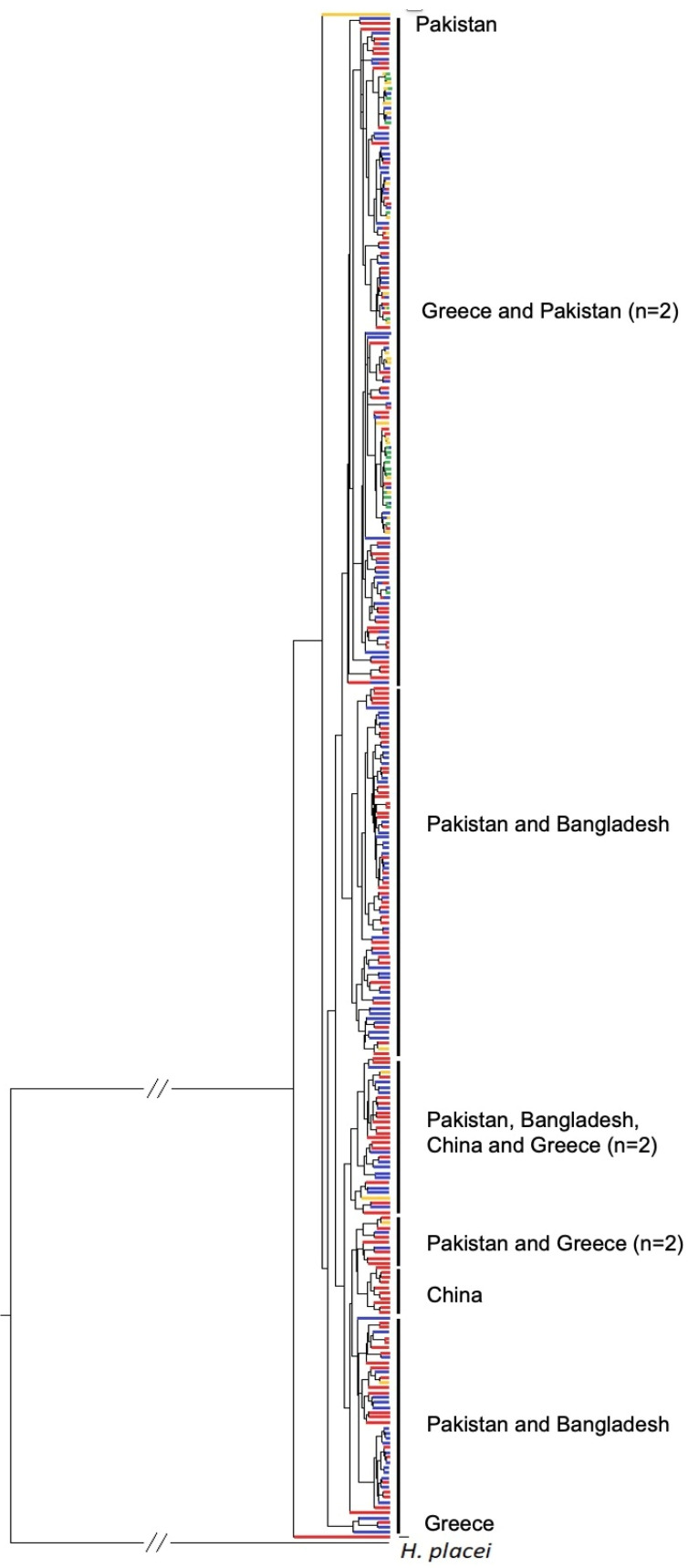
Bayesian phylogenetic tree, based on 673 bp aligned sequences (Dataset 2), of subunit 4 of the nicotinamide dehydrogenase (ND4) gene of mitochondrial DNA, which have been grouped into 306 haplotypes. The classification of the colors among four host species is as follows: red corresponds to sheep, blue to goats, yellow to cattle, and green to buffaloes.

**Figure 3:**
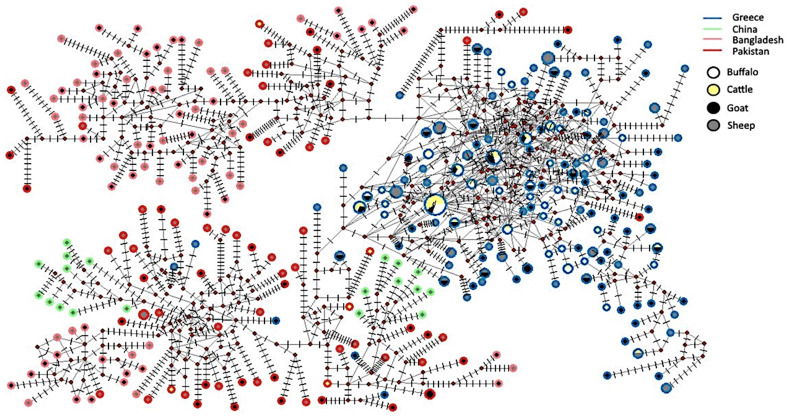
“Median-Joining” network for *H. contortus,* based on 673 bp aligned sequences (Dataset 2), of subunit 4 of the nicotinamide dehydrogenase (ND4) gene of mitochondrial DNA. The small red circles represent intermediate haplotypes, which do not correspond to certain haplotypes, in the present analysis. The vertical small-length lines are representative of the number of mutations between two haplotypes. The classification of the colors of the outlines of the big circles (i.e., refers to haplotypes’ geographical origin) is as follows: blue corresponds to Greek haplotypes, green to Chinese ones, rose to Bangladeshi ones and red to Pakistani ones. The classification of the colors of the inner part of the big circles (i.e., refers to different hosts’ species) is as follows: white corresponds to buffaloes, yellow to cattle, black to goats and grey to sheep.

Both the Bayesian phylogenetic tree (Figure 2) and the Median-Joining network (Figure 3) showed that haplotypes from different countries were grouped in separate clades. More precisely, they originated from four countries: Pakistan, Bangladesh, China, and Greece. All but five haplotypes from Greece were grouped together in a distinct clade. Two (one from sheep and one from goats) out of five haplotypes were grouped with Pakistani ones, two (both from goats) with Chinese ones, and one (from sheep) formed a separate clade. Within the Greek clade, no geographical structure was observed. Additionally, the Median-Joining network revealed haplotypes that are common among at least three different hosts and have the highest frequency (as indicated by their higher diameter), confirming that there is no clear grouping of haplotypes according to the host species from which the parasites were collected. In other words, this parasite can indiscriminately infect all species of ruminants (i.e., sheep, goats, cattle, and buffaloes), suggesting a large gene flow without clear host boundaries. In the network analyses, a “star-like” phylogeny in the Greek group was also observed in which some haplotypes seem to have emerged from an original haplotype that is the most common.

## 4. Discussion

The first aim of the present study was to molecularly identify the different *Haemonchus* species originated from four domesticated ruminant species from continental and insular Greece, based on the nuclear second internal transcribed spacer (ITS2).

The ITS2 sequence analysis confirmed the prevalence of the parasite species *H. contortus* in both large and small ruminants in Greece. This contradicts the well-known preference of different parasitic species for a particular host. To be specific, *H. contortus* prefers to infect sheep and goats, while *H. placei* prefers to infect mainly cattle [23,30,31]. In previous studies from Greece, Himonas [13], Theodoridis et al. [11], and Charalambidis [12] reported the detection of *H. contortus* in sheep and goats and *H. placei* in cattle, based on morphological criteria. Finally, there is no reference in the literature regarding *Haemonchus* spp. which infect buffaloes in our country.

Hussain et al. [23] found, through molecular approaches, that *H. contortus* was predominant in sheep and goats at a percentage of 80.9% and 91.2%, respectively, while *H. placei* was predominant in cattle at a percentage of 46.2%. Similar surveys, from Brazil and the USA, confirmed the high rate of *H. contortus* infection among small ruminant flocks [18,32]. Nevertheless, these researchers identified the presence of *H. placei* in more than 90% of cattle [18,30]. Finally, in accordance with our results, Akkari et al. [33] recorded the predominance (61.9%) of *H. contortus* in Tunisian cattle [33].

One possible explanation for the presence of *H. contortus* as the only species that infects sheep, goats, cattle, and buffaloes in Greece, is related to the management system and pasture management. *H. contortus* infects hosts through grazing. Consequently, the semi-intensive and, in some cases, extensive management systems, which are based on the grazing of animals in natural pasturelands, are mainly responsible for the infection of animals as well as the spread of this parasite in the environment [34]. At the same time, it is accepted that the grazing of a pastureland by different ruminants favors their infection with the species *H. contortus*, a situation which characterizes pasture management in Greece [32]. On the other hand, the grazing of a pastureland exclusively by cattle favors their infection with *H. placei* [32]. Therefore, in cases of common grazing of a Greek pastureland by different ruminant species, *H. contortus* is transmitted from sheep and goats to cattle and buffaloes.

The number (n = 19) of the identified ITS2 haplotypes in our study was relatively high, confirming other studies conducted in Bangladesh and China (i.e., 19 and 18, respectively). On the contrary, studies in the Helan Mountains (China), Pakistan, Brazil, and Yemen and Malaysia reported fewer genotypes (i.e., 10, 7, 6, and 6, respectively) [18,22,23,35,36]. Among the polymorphic positions detected in the present study, the variations at loci 123 and 196 were common in all previous studies, while variations at loci 59, 63, and 78 were observed at least in two countries. The variation at locus 187, which was identified in the present study, has not been reported before.

The second aim of the present study was to investigate, for the first time, the genetic diversity of *H. contortus* parasites in Greece, based on the mitochondrial nicotinamide dehydrogenase 4 (ND4).

Little is known about the population genetics of several ruminants’ parasites, although it is generally accepted that the host plays an important role in genetic diversity and gene flow between parasitic populations [14]. Some nematode species create large pools of genes in different geographical areas, and this is attributed, at least in some cases, to the widespread movement of their hosts.

The nucleotide diversity of the Greek *H. contortus* population is marked by a wide range varying from 0.007 to 0.021, with an average value of 0.017. The recorded nucleotide diversity in the present study is relatively low compared to published values from other countries such as Italy (0.026–0.030) [37], Malaysia (0.032–0.044) [35], the USA (0.024–0.030) [38], Yemen (0.021–0.036) [35], and China (0.018–0.037) [36]. The average haplotype diversity (0.995) of the Greek *H. contortus* population is slightly lower compared to the respective results from other studies. More precisely, Hussain et al. [23] and Shen et al. [24] recorded a 0.998 mean haplotype diversity of *H. contortus* population, originated from 40 sheep, 30 goats, 6 cattle, and 1 buffalo, and 0.999, originated from 5 blue sheep (i.e., non-domesticated sheep) and 6 sheep, respectively. According to Yin et al. [36], the haplotype diversity ranged between 0.995 and 1.000 in six out of seven studied regions, while in one region, the recorded value was lower (i.e., 0.993) than ours.

The size of the parasitic population significantly contributes to genetic diversity [39]. Nematodes of the genus *Haemonchus* are characterized by high fertility, which in combination with its high infectious capability and its direct life cycle (no intermediate host is involved), contribute to the rapid increase of the parasitic population and its wide spreading [40]. These are enhanced in cases of the coexistence of sheep and goats, not only in the same farms but also in common pasturelands, contributing to the wide spreading of these parasites among ruminants belonging to the same or different species. For the aforementioned reasons, a large gene flow takes place between *H. contortus* individuals, which justifies the high nucleotide and haplotype diversity of the parasitic population of small ruminants in Greece.

An important question that arises is whether the high genetic diversity, which is observed in the population of these parasites of small ruminants in Greece, depends on the migration of genotypes from other populations. Despite the fact that the movement of animal populations from one region to another has been historically proven, there are no studies that have found clear discontinuities in the phylogenetic relationships of mitochondrial ND4 haplotypes of *H. contortus*, indicating little or no movement of their hosts [41,42,43]. This was confirmed in our study, since 135 out of 140 haplotypes were grouped together in a distinct clade. However, this issue would be better understood, if more sequences from other countries could be included in our analysis.

The nucleotide and haplotype diversity of *H. contortus* populations originated from large ruminants is lower compared to the respective values from small ruminants. Regarding cattle populations, these low values can be attributed to the decreased percentage of *H. contortus* infection, which is directly related to the management system, as the majority of cattle are intensively reared and only a small percentage of them are reared according to the semi-intensive system, which allows grazing in natural pasturelands. In addition, the strong immune response of cattle against this parasitic species contributes to the low infection rate of cattle, as a higher number of *H. contortus* is required for the clinical appearance of symptoms [44]. The nucleotide and haplotype diversity of *H. contortus* populations from buffaloes is evaluated for the first time universally, to the best of our knowledge. The lower nucleotide and haplotype diversity in buffaloes, compared to the respective ones in small ruminants, is probably due to the limited population of buffaloes in Greece and, consequently, to the small number of samples collected [45,46]. The low nucleotide diversity, combined with the high haplotype diversity, in buffaloes’ parasitic populations indicates a high number of closely related haplotypes and suggest that this population may have undergone a recent expansion [44,45,46].

A considerable number of unique haplotypes infecting only sheep and/or goats were found at a percentage of 64.9% and 66.7%, respectively. One possible explanation is related to the sample size. In other words, the high genetic diversity of *H. contortus* populations of small ruminants in combination with the number of samples used in the molecular analysis contributed to this increased percentage of unique haplotypes. In recent decades, the control of *H. contortus* has strongly relied on anthelmintics, the most used of which are albendazole. Previous studies conducted in Greece revealed a high frequency of the homozygous benzimidazole-resistant alleles in *H. contortus* helminths from small ruminants [47]. As the level of gene flow among populations of *H. contortus* parasitizing sheep and goats in Greece was shown, in the present study, to be very high, there is substantial opportunity for these resistant alleles to spread. This fact, combined with the different patterns of metabolism of the anthelmintics between sheep and goats, could explain the high number of unique haplotypes recorded in the present study. Another possible hypothesis justifying the existence of unique haplotypes in small ruminants is the special terrain of Greece in combination with the different management systems and grazing behavior of sheep and goats. These seem to have created the right conditions for the survival and evolution of different parasitic populations in each ruminant species. This hypothesis has been supported by Cerutti et al. [37], who recorded a higher genetic variability in the alpine context, which includes various management systems, different breeds and host species, and climatic and geographical barriers, than in North America.

The phylogenetic analysis of ND4 gene did not reveal a clear grouping of the haplotypes with their hosts. Troell et al. [15] and Cerutti et al. [37] confirmed our result during the phylogenetic analysis of the same gene from alpine wild goat, deer, and chamois, as well as from domestic sheep and goats. On the contrary, Gharamah et al. [35] concluded that during the phylogenetic analysis of the mitochondrial ND4 gene, a distinct group of sequences was created, which originated exclusively from *H. contortus* of goats in Yemen [35]. This conclusion demonstrated the possibility of a parasitic subpopulation, which can infect only goats and not sheep in Yemen.

The genetic analysis of the ND4 gene, in the present study, showed that the majority (i.e., 135 out of 140) of Greek haplotypes were grouped together with no clear geographic structuring, suggesting a high gene flow without clear geographical barriers among *H. contortus* populations from different parts of Greece. An exception was a subset of five Greek haplotypes, originated from small ruminants (i.e., two sheep and three goats), which were grouped together with Asian strains of Chinese, Pakistani, and Bangladeshi origin. This phylogenetic relationship was not surprising, since several diseases of veterinary importance follow the same path to Greece [48]. The two common pathways are through the Aegean islands, adjacent to the coast of Anatolian Turkey or through the north-eastern prefecture of Evros, adjacent to the Turkish and Bulgarian borders. The genetic relationship between Greek, southeast Asian, and Australian *H. contortus* has been observed by Troell et al. [15], suggesting the transport of these nematodes, presumably in infected hosts, by European colonizers.

From the network analysis, a clear grouping of the Greek haplotypes of the *H. contortus* population was obvious. This result showed a low genetic relationship of the Greek with the Asian parasitic strains which was, further, attributed to no or low importation of ruminants (hosts of *H. contortus*) from the Asian territory to our country. The close phylogenetic relationships of the Greek haplotypes suggested a lack of grouping of the Greek haplotypes based on their geographical origin, in agreement with previous studies conducted in China [24,36]. Another finding of the present phylogenetic analysis was the lack of grouping of *H. contortus* according to host species, which in combination with no clear geographic structuring, supported the massive movements of their hosts in the Greek territory. These movements, accompanied with co-grazing in common fields, led to the spread of this helminth among different ruminant species and to the wide exchange of the genes among *H. contortus* populations; thus, it contributed to the high genetic diversity of this parasitic population in Greece. This result is similar to findings of previous studies showing a limited relationship of *H. contortus* populations with different ruminant species in Brazil [18], Italy [37], Malaysia and Yemen [35], and China [24,36]. Finally, the “star” form of the network reinforces the idea of the rapid spread and colonization of *H. contortus* nematodes in Greece, in which most haplotypes seem to have emerged from an original haplotype which is the most common.

## 5. Conclusions

The present study identified, for the first time using molecular approaches, the presence of *H. contortus* as the exclusive species of the genus *Haemonchus* infecting sheep, goats, cattle, and buffaloes in continental and insular Greece. Moreover, the present study is the first to genetically characterize *H. contortus* specimens isolated from small and large ruminants in Greece. A novelty of the present study is the fact that small ruminants raised on mixed farms were included, a practice that does not exist abroad. Based on the results of the phylogenetic analysis, a large genetic diversity of *H. contortus* was recorded regardless of the host species and geographical origin within Greece. This diversity is attributed to the cross-infection between sympatric species of ruminants in Greece mainly due to common grazing.

## Figures and Tables

**Table 1:** Primers used and product size after the amplification of the internal transcribed spacer 2 (ITS2) of nuclear DNA and subunit 4 of nicotinamide dehydrogenase (ND4) of mitochondrial DNA, of *Haemonchus* spp.

Primer	Sequence	Product Size (bp)
NC1-F	forward: 5′- ACGTCTGGTTCAGGGTTGTT -3′	321
NC2-R	reverse: 5′- TTAGTTTCTTTTCCTCCGCT -3′	
OP1-F	forward: 5′- GGATTTGGTCAGCAAATTGAA -3′	820
OP2-R	reverse: 5′- TCATTTGTGGTTACCTAAAGC -3′	

NC1-F: forward primer for the amplification of ITS2; NC2-R: reverse primer for the amplification of ITS2; OP1-F: forward primer for the amplification of ND4; OP2-R: reverse primer for the amplification of ND4.

**Table 2:** Information (accession numbers, number of sequences, hosts and their origin, references, and dataset) for the GenBank-retrieved sequences used for the phylogenetic analysis of subunit 4 of the nicotinamide dehydrogenase (ND4) mitochondrial DNA gene. Dataset 2 includes all Greek sequences plus 169 sequences with 673 bp overlapping regions.

Accession Numbers	Number of Sequences	Hosts	Origin	Reference	Dataset
LC361049–LC361102, LC376827–LC376849	77	Sheep & goats	Bangladesh	Dey et al. [22]	2
KJ724439–KJ724511	73	Sheep, goats & cattle	Pakistan	Hussain et al. [23]	2
KY305790–KY305808	19	Sheep	China	Shen et al. [24]	2

**Table 3:** Nucleotide details and frequency distribution of second internal transcribed spacer (ITS2) genotypes of 191 parasites isolated from four hosts (i.e., buffaloes, cattle, goats, and sheep).

Genotype	Position						Number of Parasites				
	59	63	78	123	187	196	B	C	G	S	Total
X78803	T	C	T	C	G	A	-	-	-	-	-
GT1	.	.	.	T	G	T	3 ^a^	3 ^a^	17 ^b^	14 ^b^	37
GT2	.	.	.	T	R	T	25 ^a^	28 ^a^	17 ^b^	25 ^a^	95
GT3	.	.	.	T	R	W	1 ^a^	1 ^a^	2 ^b^	-	4
GT4	.	.	.	T	.	W	1 ^a^	-	1 ^a^	-	2
GT5	.	.	.	Y	.	W	-	3 ^a^	4 ^b^	2 ^a^	9
GT6	.	.	Y	Y	.	T	-	-	3	-	3
GT7	.	.	Y	Y	.	W	-	1 ^a^	2 ^a^	4 ^b^	7
GT8	.	.	Y	T	.	T	-	-	1 ^a^	3 ^a^	4
GT9	.	.	.	Y	.	T	1 ^a^	-	2 ^a^	-	3
GT10	.	.	Y	Y	R	W	-	1 ^a^	2 ^a^	2 ^a^	5
GT11	.	Y	Y	Y	R	W	-	-	1	-	1
GT12	.	.	.	Y	R	W	1 ^a^	-	2 ^a^	1 ^a^	4
GT13	K	Y	Y	Y	.	T	4 ^a^	1 ^b^	2 ^b^	2 ^b^	9
GT14	.	.	Y	T	R	T	-	-	1	-	1
GT15	K	Y	.	Y	.	W	1 ^a^	-	1 ^a^	-	2
GT16	K	Y	Y	Y	R	W	1 ^a^	-	1 ^a^	-	2
GT17	.	Y	.	Y	.	W	-	1	-	-	1
GT18	.	.	.	Y	R	T	1	-	-	-	1
GT19	.	Y	Y	Y	.	W	-	-	-	1	1
Total							39 ^a^	39 ^a^	59 ^b^	54 ^b^	191

B: buffalo; C: cattle; G: goat; S: sheep. X78803: *Haemonchus contortus*. Dots represent identical nucleotide positions. ^a, b^ different superscripts in each row indicate statistically significant differences in the distribution of the frequencies of the ITS2 genotypes among the ruminant species.

**Table 4:** Genetic diversity values for *H. contortus* parasites isolated from four different hosts (i.e., buffaloes, cattle, goats, and sheep).

Ruminants	Dataset	Base Pairs (bp)	Ν	H	Hp	V (%)	W (%)	Hd (SD)	Nd (SD)
Buffaloes	1	690	24	22	13	91.7	54.2	0.989 ^a^ (0.017)	0.007 ^a^ (0.001)
	2	673	-	-	-	-	-	-	-
Cattle	1	690	33	25	14	75.8	42.4	0.966 ^b^ (0.022)	0.007 ^a^ (0.001)
	2	673	38	30	18	78.9	47.4	0.974 ^A^ (0.017)	0.012 ^A^ (0.002)
Goats	1	690	63	61	42	96.8	66.7	0.999 ^c^ (0.003)	0.019 ^b^ (0.001)
	2	673	134	131	112	97.8	83.6	0.999 ^B^ (0.001)	0.029 ^B^ (0.001)
Sheep	1	690	74	59	48	79.7	64.9	0.997 ^c^ (0.003)	0.021 ^c^ (0.001)
	2	673	167	151	140	90.4	83.8	0.999 ^B^ (0.001)	0.029 ^B^ (0.001)
Total samples	1	690	194	140	117	72.2	60.3	0.995 ^c^ (0.002)	0.017 ^b^ (0.001)
	2	673	363	306	282	84.3	77.7	0.998 ^B^ (0.001)	0.026 ^B^ (0.001)

N, sample size; H, number of different haplotypes; Hp, host specific haplotypes; V, the percentage of the total number of haplotypes/number of individuals; W, the percentage of the host specific haplotypes/number of individuals; Hd, haplotype diversity; Nd, nucleotide diversity; SD, standard deviation. ^a, b, c^ and ^A, B^ different superscripts in each column indicate statistically significant differences of the haplotype (Hd) and nucleotide diversity (Nd), among the ruminant species, for Dataset 1 and 2, respectively.

## Data Availability

All data are included into the manuscript.
